# Mathematical Modelling of Nitric Oxide/Cyclic GMP/Cyclic AMP Signalling in Platelets

**DOI:** 10.3390/ijms19020612

**Published:** 2018-02-19

**Authors:** Rune Kleppe, Inge Jonassen, Stein Ove Døskeland, Frode Selheim

**Affiliations:** 1 Computational Biology Unit, Department of Informatics, University of Bergen, Thormøhlensgate 55, 5008 Bergen, Norway; inge.jonassen@uib.no; 2 Department of Biomedicine, Proteomics Unit at University of Bergen (PROBE), University of Bergen, Jonas Lies vei 91, 5020 Bergen, Norway; Stein.Doskeland@uib.no (S.O.D.); Frode.Selheim@uib.no (F.S.)

**Keywords:** nitric oxide, platelets, cGMP signalling, cAMP signalling, mathematical modelling, phosphodiesterase, PDE2, PDE3, PDE5

## Abstract

Platelet activation contributes to normal haemostasis but also to pathologic conditions like stroke and cardiac infarction. Signalling by cGMP and cAMP inhibit platelet activation and are therefore attractive targets for thrombosis prevention. However, extensive cross-talk between the cGMP and cAMP signalling pathways in multiple tissues complicates the selective targeting of their activities. We have used mathematical modelling based on experimental data from the literature to quantify the steady state behaviour of nitric oxide (NO)/cGMP/cAMP signalling in platelets. The analysis provides an assessment of NO-induced cGMP synthesis and PKG activation as well as cGMP-mediated cAMP and PKA activation though modulation of phosphodiesterase (PDE2 and 3) activities. Both one- and two-compartment models of platelet cyclic nucleotide signalling are presented. The models provide new insight for understanding how NO signalling to cGMP and indirectly cAMP, can inhibit platelet shape-change, the initial step of platelet activation. Only the two-compartment models could account for the experimental observation that NO-mediated PKA activation can occur when the bulk platelet cAMP level is unchanged. The models revealed also a potential for hierarchical interplay between the different platelet phosphodiesterases. Specifically, the models predict, unexpectedly, a strong effect of pharmacological inhibitors of cGMP-specific PDE5 on the cGMP/cAMP cross-talk. This may explain the successful use of weak PDE5-inhibitors, such as dipyridamole, in anti-platelet therapy. In conclusion, increased NO signalling or PDE5 inhibition are attractive ways of increasing cGMP-cAMP cross-talk selectively in platelets.

## 1. Introduction

**Figure 1:**
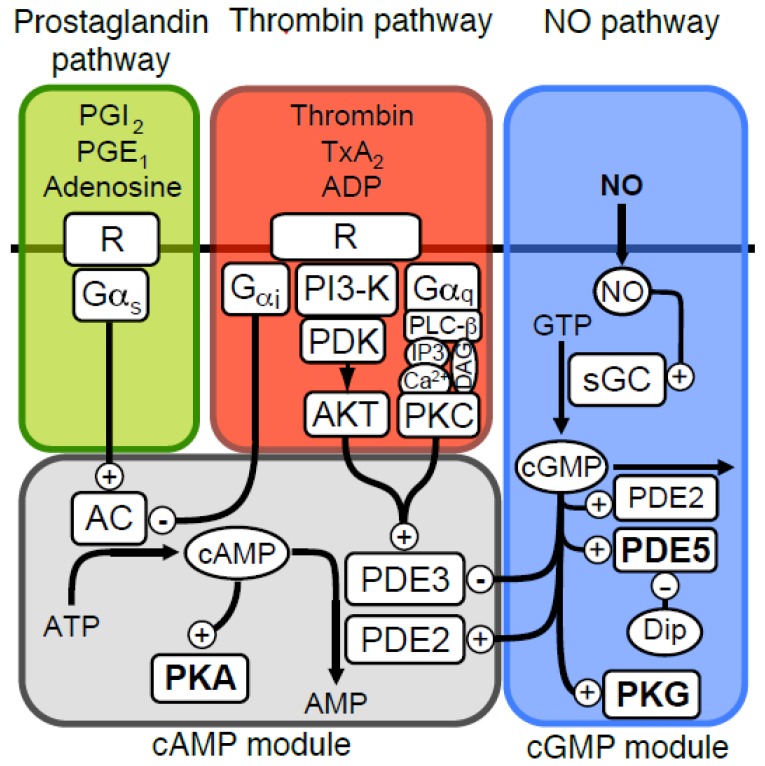
Signalling pathways affecting platelet cyclic nucleotides. An overview of the platelet signalling pathways which influence cGMP and cAMP levels are shown; nitric oxide (NO) stimulate cGMP production by activating soluble guanylate cyclase (GC). The cGMP signal is degraded by phosphodiesterases (PDEs), where PDE5 dominates and PDE2 contributes slightly. The cGMP dependent protein kinase is activated by cGMP and executes much of the signalling by phosphorylating important target proteins. The cGMP module also mediates cross-talk with the cAMP-module through cGMP production that stimulates (PDE2) or inhibits (PDE3) cAMP phosphodiesterases. cAMP is produced by adenylate cyclase (AC, EC 4.6.1.1) that is activated by Gα_s_ stimulation, elicited by receptor binding of ligands such as prostaglandins (PGI_2_, PGE_1_) or adenosine. Platelet agonists such as thrombin, ADP and thomboxane (TxA_2_) will inhibit cAMP signalling, by inhibiting AC through inhibitory Gα_i_, or by increased cAMP degradation through phosphorylation-mediated activation of PDE3 by AKT/PKB or PKC.

Platelets play an important role in the haemostatic machinery, by responding rapidly to damage of the vascular wall. This response is mediated through several signalling pathways acting downstream of receptors for von Willebrand Factor (vWF), thrombin and collagen. Autocrine stimulatory factors such as ADP and thromboxane are important for stimulating further adhesion to fibrinogen, vWF and collagen (through integrin receptors α_IIb_β_3_, α_2_β_1_) and for activating circulating platelets. The self-stimulatory nature of this process necessitates strict negative regulation. This is achieved mainly through endothelial factors such as prostacyclin (PGI_2_) and nitric oxide (NO) that activate cAMP and cGMP signalling pathways [1] (Figure 1). Deficient inhibitory signal communication from the endothelium is associated with severe vascular disorders such as atherosclerosis and acute ischemic stroke [2].

NO rapidly stimulates cGMP production in platelets, by binding to the heme moiety of the soluble guanylyl cyclase (sGC) (EC 4.6.1.2) [1]. Several downstream targets have been characterized for this important inhibitory pathway including the cGMP-dependent protein kinase (PKG, EC 2.7.11.12) and its substrates vasodilator stimulated phosphoprotein (VASP), inositol 1,4,5-trisphophate receptor-associated cGMP-dependent protein kinase (IRAG) and Ras-related protein (Rap1B) [1,3]. The second messenger cGMP is rapidly degraded by phosphodiesterases (EC 3.1.4.17), in particular the cGMP-specific phosphodiesterase 5A (PDE5), which is abundantly expressed in platelets [4] (Figure 1). PDE5 is activated by binding of cGMP to one of its N-terminal GAF (cGMP-specific and -stimulated, *Anabaena* adenylate cyclase and *Escherichia coli* FhlA) domains, which increases both its *V*_max_ activity and its affinity for cGMP (lowers the *K*_m_-value) [5,6]. In addition, PKG can sensitize PDE5 to cGMP by phosphorylation, leading mainly to increased affinity of cGMP to the activating GAF-domain [7].

We have previously shown that NO inhibits platelet shape change through activation of cAMP-dependent protein kinase (PKA, EC 2.7.11.11) [8]. Platelet shape change is a reversible process that involves reorganization of the cytoskeleton, resulting in a transition from discoid to spherical shape with extending pseudopodia that facilitates adhesion to the vessel wall and thrombus formation. Cross-communication between cGMP and cAMP has been reported in many tissue and cell types ([9,10,11,12]) and can be inhibitory or stimulatory depending on the components involved. In platelets [13] the cross-talk is mostly mediated through the cGMP-stimulated cAMP degradation by phosphodiesterase 2A (PDE2) and cGMP-inhibited cAMP degradation by phosphodiesterase 3A (PDE3) (Figure 1). No cross-communication from cAMP to cGMP has been reported in platelets [14], although PKA can phosphorylate PDE5 on the PKG site (Ser92), but with 10-fold lower efficiency than PKG [6].

Nitric oxide stimulation of platelets gives zero to very moderate changes in total cAMP levels [15,16]. Furthermore, inhibitors of cyclic nucleotide phosphodiesterase PDE3 are more efficient than PDE2 inhibitors to inhibit thrombin-induced platelet shape change and secretion, although PDE2 inhibition gives rise to higher total cAMP levels [8,13]. This suggests that compartmentalized cAMP signalling may be important for regulating shape change in platelets.

Clearly, the development of unfavourable thrombotic events rely on complex relationships between platelets themselves and the surrounding mechano-chemical environment. A more quantitative understanding of platelet behaviour at different conditions would expectedly facilitate the identification of high-risk situations and the appropriate treatment. Here we investigated the cross-communication between platelet cGMP/cAMP (cNMP) pathways downstream of NO by mechanistic modelling. We studied the role of PDE5 in this cross-talk in details, as PDE5 inhibitors such as dipyridamole, are used in the clinic together with aspirin for the secondary prevention of stroke [17]. We have previously shown that NO and dipyridamole act synergistically to inhibit platelet shape change without increasing the cAMP levels [15]. To understand the underlying mechanism for this observation we performed modelling of platelet cGMP and cAMP signalling in both one- and two compartments to assess how different degrees of compartmentation of AC and PDEs influence the signalling cross-talk between cGMP and cAMP. We show that only a two-compartment model can explain the experimentally observed cyclic nucleotide concentrations.

## 2. Results

### 2.1. The Modelling Approach and Aims

We have applied a mechanistic modelling approach where ordinary differential equations are used to describe experimentally derived mechanistic and kinetic descriptions of the molecular components of platelet cGMP and cAMP signalling (Figure 1). This is a common approach and has been used in several previous studies on platelets [14,18,19]. The proteins involved in the signalling pathways are well described regarding the equilibrium binding affinities and the enzyme kinetic constants, although many of the rate constants are less well elucidated. We have therefore mainly focused on the steady state behaviour of the model. The modelling was undertaken in part to be able to predict the impact of perturbations (normal or pharmacological) on the activity of the cGMP/cAMP signalling cascades. The models should quantify contributions to the signalling process, coming from different molecular activities and depending on concentrations of key components in platelets. Within these frames we further investigated possible mechanisms for compartmentalized signalling, relevant to understand NO/cGMP mediated cAMP/PKA activation. Signal compartmentalization has been reported experimentally in platelets [20] and we wanted to investigate how different compositions of the signalling components would affect the signalling response in a cyclic nucleotide signalling compartment.

### 2.2. A Steady State Model of the NO-cGMP-PKG Pathway

The pre-steady state NO-induced cGMP formation has been investigated in several studies [4,21,22]. Under such conditions where NO stimulation has been lacking during in vitro platelet preparation, a pulsed cGMP response is observed prior to relaxation to steady state levels. We here focus on the steady state level of cGMP, as platelets are under constant exposure to NO in vivo. The activation of soluble guanylyl cyclase (sGC) was modelled as described (Materials and Methods), relying mainly on the quantitative measurements of sGC and cGMP-PDE activities performed on human platelets (Table 1, [4]).

The cGMP produced can bind to the two cGMP binding sites of PKG, the GAF-domains of PDE5 and PDE2, and the active site of PDE3. The level of the PKG receptor is very high levels in human platelets (7.3 µM) [33], giving 14.6 µM cGMP binding sites from this receptor alone. The levels of PDE2, 3 and 5 have also been reported but the high estimates found using quantitative western blotting was not consistent with immunostaining of platelets [14] or mass spectrometric measurements [18]. The modelling performed in those studies relied on only a fraction of platelets PDEs being present in a catalytically competent state. We have therefore not included cGMP bound to the GAF-domains of PDE5 or PDE2 or to the active site of PDE3 when calculating total cGMP levels. Such binding, in particular the tight binding to the PDE5 GAF-A domain, could indeed influence the measured cGMP level, depending on the experimental method used.

**Figure 2:**
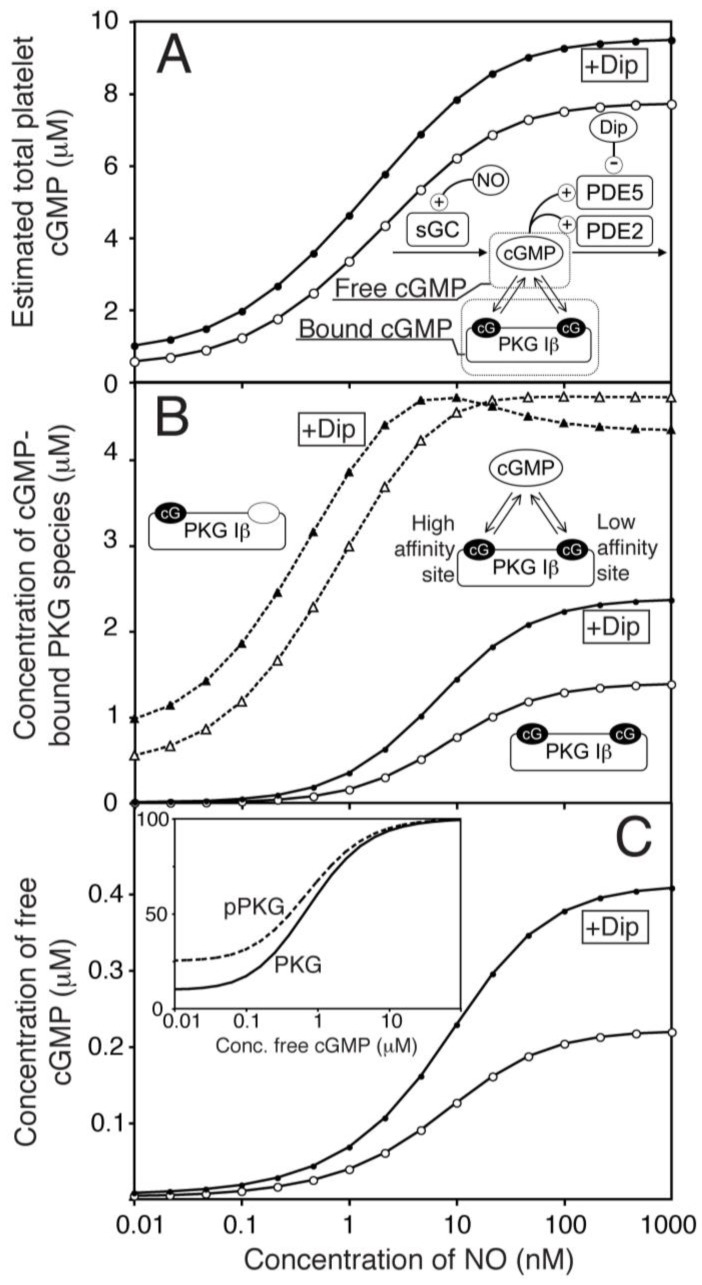
One-compartment model of platelet NO/cGMP signalling. (**A**) The total concentrations of cGMP in platelets (bound & free) were estimated by mechanistic modelling in response to different NO concentrations, in the absence (○) or presence of dipyridamole (●, 1.0 μM). The key molecules that determine free and bound cGMP-levels are illustrated in the reaction network. (**B**) shows the computed levels of cGMP-bound PKG Iβ at different NO concentrations in the absence (open symbols) and presence (closed symbols) of dipyridamole. Binding of cGMP to the high-affinity site (△,▲, dotted line) and both sites (○,●, solid lines) are shown. (**C**) shows the predicted concentration of free cGMP at different NO concentrations in the absence (○) or presence of dipyridamole (●). The inset shows predicted PKG activity at different cGMP concentrations for non-phosphorylated (solid line) and completely autophosphorylated PKG (pPKG, dotted line).

The present platelet cGMP model differs from previous models [14,18,21,34] by considering the impact of cGMP binding to cGMP receptors, like PKG, which has an unusually high expression in platelets (7.3 µM) and has been found experimentally to protect cGMP from PDE5 mediated degradation [30]. Such sequestration decreases the concentration of free cGMP available for degradation by PDE5, allowing the model to reach the experimentally determined steady state cGMP-level at conditions with higher PDE5 *V*_max_ than in absence of sequestration. The basal platelet cGMP level is reported to be in the low microM range (0.45 µM). This value is in the range of the binding constant (*K*_D_ 0.75 µM) of the low affinity site in platelet PKG Iβ [30]. However, a basal cGMP level (total conc.) at that level corresponds to a free cGMP concentration 100 fold less. This low basal cGMP level could only be obtained with a basal GC activity that was very low (1/400 of maximal), which is close to measurements made on the purified recombinant GC enzyme [24]. Using NO-donors, a maximal steady state cGMP level of ~7.6 µM [16] was obtained, which we have used as maximal total cGMP-concentration in our model (Figure 2A). This gives an NO-response curve that is sensitive to low NO-concentrations, in concordance with physiologically realistic values (0.1–5 nM [35]). It is worth mentioning that the half maximal response is considerable lower than that of the GC (11 nM NO), probable due to the large buffer effect of the PKG high affinity site (Figure 2B), as half maximal free cGMP is at NO concentrations closer to the half maximal GC activation (Figure 2C).

### 2.3. Effects of the Moderate PDE5-Inhibitor Dipyridamole on cGMP Levels

We have previously shown that the moderate PDE5 inhibitor dipyridamole does not affect thrombin-induced shape change in the absence of NO but prolong the NO-mediated inhibition of platelet shape change [15]. By including dipyridamole in the model as a competitive PDE5 inhibitor (*K*_i_ = 0.7 µM), we could test whether PDE5 inhibition alone could explain our previous experimental observations and estimate the effect of dipyridamole at all NO levels (Figure 2A). We found that dipyridamole (1.0 µM) increased the total cGMP level at NO stimulation by 23% (at maximal NO), close to our experimental observations (24%).

The model was also used to estimate the effect of dipyridamole on PKG activation. Notably, the rather low maximal cGMP-level in platelets compared to the high level of PKG gave a low maximal activation of this kinase (19%) and a sluggish response to NO (Figure 2B). However, dipyridamole is predicted to have a larger impact on PKG activation (>72% Figure 2B,C) than expected from the 23% increase in total cGMP. This can be more easily understood from the dipyridamole-induced changes in free cGMP, which were roughly doubled (Figure 2C).

### 2.4. PKG-Mediated PDE5 Phosphorylation Has Little Influence on the Steady State NO-cGMP Response Curve

**Figure 3:**
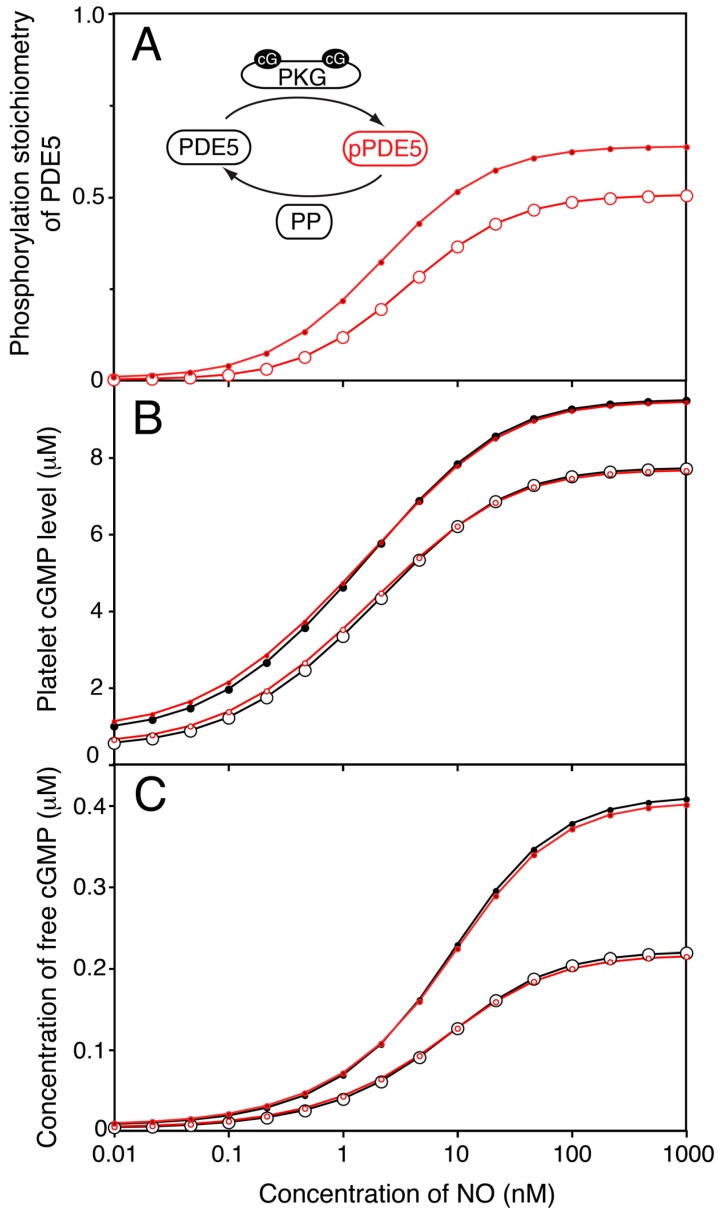
Effect of PKG mediated phosphorylation of PDE5 on cGMP response to NO. Assuming a maximal 50% phosphorylation of PDE5 in maximally NO stimulated platelets, we calculated the impact of this phosphorylation by PKG on the NO-stimulated cGMP response curve. (**A**) shows the phosphorylation stoichiometry of PDE5 at different concentrations of NO in the absence (○) or presence (●) of dipyridamole (1 µM). In (**B**,**C**) we compare the model with (in red) and without (in black) phosphorylation of PDE5 included and the impact on total platelet cGMP levels (**B**) or concentration of free cGMP (**C**). The model without PDE5 phosphorylation is shown in black, pPDE5-model in red. Open and closed symbols denote absence and presence of dipyridamole, respectively. For both models, the *V*_max_ of PDE5 was fitted to the same maximal and basal levels of total cGMP.

Phosphorylation is reported to affect PDE5 and 3 activities in platelets. For PDE5, phosphorylation seems to mainly affect binding of cGMP to the GAF-A domain, lowering the *K*_D_ from 130 to 30 nM [7], which is expected to give a slightly steeper cGMP response curve to NO. We also included a 20% increased *V*_max_ by phosphorylation but since the model is adjusted for experimentally determined cGMP levels, this had only minor effect on the model behaviour. For rat platelets the phosphorylation stoichiometry of PDE5 was found to remain unchanged during NO stimulation [34], whereas it has been found to increase in human platelets, however not quantified [4]. We wanted to investigate the impact of a relatively large increase in PDE5 phosphorylation, rising to 50% at maximal NO, on the cGMP response curve (Figure 3A). In spite of having a 150 fold increase in phosphorylation, the impact was very modest on both total cGMP- and free cGMP-levels (Figure 3B,C). This fits with observations on the effect of PDE5 phosphorylation of human platelet cGMP response to NO [36]. As expected, dipyridamole increased the maximal PDE5 phosphorylation but this had only minor effects on the cGMP levels compared to the model without PDE5 phosphorylation (Figure 3A–C). Possibly, the PKG-mediated PDE5 phosphorylation is functionally implicated in the dynamics of platelet function, rather than the steady state responses. Thus, a more than 4 fold increase in affinity for the cGMP-activation site is expected to influence the platelet response to subsequent stimuli during their flow in the blood stream.

### 2.5. Modelling the Influence of cGMP on Platelet cAMP Levels through PDE3

Nitric oxide does not activate platelet adenylate cyclase (AC). Furthermore, inhibitors of PDE3 but not of PDE2, can mimic the inhibitory effect of NO on platelet shape change [8]. We therefore first studied the activity of PDE3 in response to cGMP and cAMP. PDE3 is competitively inhibited by cGMP, effectively shifting its half maximal activation by cAMP (Figure S1A). As the AC activity is constant in NO stimulation and at different cAMP concentrations, the steady state level of free cAMP as a function of a given AC activity (basal: V^AC^_basal_) or stimulated: V^AC^_stim_) and PDE3 activity (V^PDE3^) for different cGMP concentrations, is given by the cAMP concentrations where the two activities are equal (Figure S1A, blue or red circles).

Increasing cAMP levels will bind to and activate platelet PKAs. The PKA isoform or isotype composition of platelets is not completely resolved. We therefore applied kinetic data from both human regulatory subunit Iα and IIα with purified bovine heart catalytic subunit (C) (Table 1, [31,32]). As the tetrameric PKA holoenzyme is symmetric, we modelled it as a heterodimer of a regulatory subunit (R) and catalytic subunit (C). The activation of PKA was modelled by sequential binding of two cAMP ligands to the R-subunit, with subsequent dissociation of the cAMP-bound holoenzyme complex as shown (Table 1, Figure S1B). The modelled response is for PKA at the level reported in platelets (3.1 µM) giving a maximal of almost 1.2 µM (39%) free C-subunit at saturating cAMP concentrations. For the cAMP pathway, considerable amounts of PKA-bound cAMP were predicted, i.e., for 10 µM free cAMP, total platelet cAMP of almost 16 µM was predicted, with about 1 µM free PKA catalytic subunit levels (Figure S1B).

Phosphorylation and activation of PDE3 by PKA is a well-known mechanism for feedback-inhibition. For illustrations, the effect of an assumed PKA-mediated PDE3 phosphorylation of maximal 90% was modelled (Figure S1C) with the concurrent alterations in the PDE3 activity (Figure S1D). Such a strong feedback regulation gave a clear reduction of the cAMP steady state response to increased cGMP levels, but affected the steady state cAMP level in response to increased AC activation more. Thus, the cAMP increase upon cGMP stimulation (to 200 nM) now became similar to that obtained by a 5-fold increase in AC activity (Figure S1D, circles 1 and 2, red versus black).

### 2.6. Modelling Global Platelet cGMP to cAMP Cross-Talk

It is well known that platelet PDE2 contributes extensively to the control of cAMP levels [37]. The PDE2 kinetics is complex due to its dual specificity in the catalytic domain and that both cGMP and cAMP can bind to its GAF domain and activate the enzyme. Multiple binding reactions to activating GAF domain and Michaelis Menten equations were used to describe the behaviour of this enzyme in concordance with experimental observations. This also gave a better fit to experimental data on PDE2 purified from human platelets than existing models (Table 1, Figure S2).

**Figure 4:**
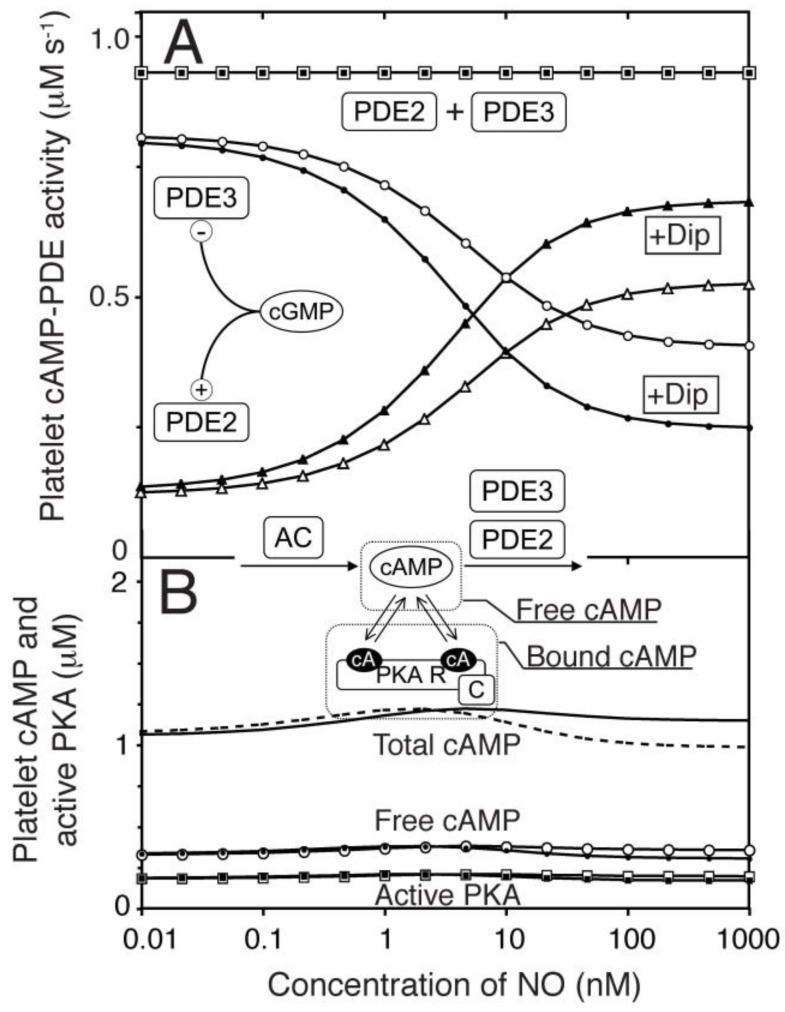
One compartment model of platelet NO/cGMP to cAMP/PKA signalling. We extended the model of the NO/cGMP signalling pathway to also include the relevant cAMP signalling components and adjusting the enzyme activities to reach experimentally observed cAMP levels at basal and NO stimulated conditions. (**A**) the predicted change in PDE2- (△▲), PDE3- (○,●) and total cAMP-PDE- (☐,■) activity was estimated in response to NO in absence (open) and presence (closed) of dipyridamole. In (**B**) we show the cAMP-response to NO for total cAMP (solid line without dipyridamole, dotted line with dip), free cAMP (○,●) and active PKA (☐,■). Open and closed symbols denote absence or presence of dipyridamole (1.0 μM), respectively.

To model the cAMP response to different NO concentrations the model was expanded to include AC as well as PDE2 and PDE3. A constant basal AC activity (<1 µM^−1^ s^−1^ [38]) was set to balance the cAMP-PDE activity at the cAMP concentration found experimentally. Similarly as for cGMP, cAMP association to PKA was included here as described (Figure S1). Others and we have previously reported very modest total cAMP changes upon NO stimulation of platelets [15,16]. This was used to fit the relative levels of PDE2 and 3 activities (Figure 4A) and our model accurately predicted the measurable changes in the NO-cAMP response by dipyridamole (1.0 µM).

PDE3 and PDE2 have different sensitivities towards cGMP and very different kinetic characteristics. It was therefore surprising that similar cAMP levels between none and high NO levels led to very small alterations in NO-mediated cAMP responses across the whole range (Figure 4A). Thus, the activities of PDE2 (cGMP-stimulated PDE) and PDE3 (cGMP-inhibited PDE) are balanced throughout the range of NO concentrations and in the presence of both NO and dipyridamole. Evidently, the total cAMP-PDE activity was unresponsive to both cGMP and dipyridamole. This model could therefore not explain the mechanism behind NO-mediated PKA activation. However, such a global model is a good starting point for investigating NO-mediated PKA activation in signal compartments where the distribution of signalling proteins could vary.

### 2.7. Two-Compartment Modelling of cGMP/cAMP Crosstalk through PDE3

**Figure 5:**
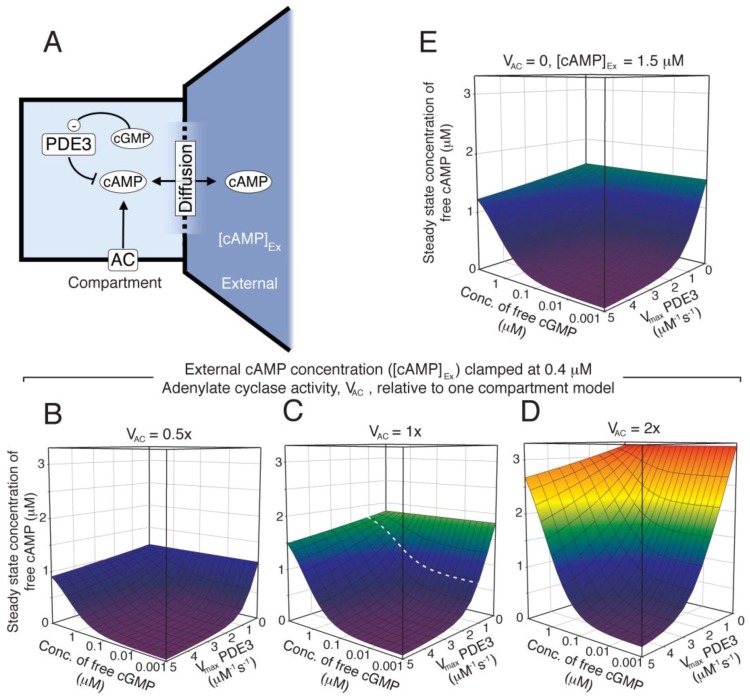
The kinetic properties of PDE3 facilitates cAMP-compartment signalling in absence of AC activation. (**A**) We investigated the formation of a steady state difference in cAMP concentration between a small compartment that were diffusion coupled to an external large compartment, that was clamped at a fixed cAMP concentration ([cAMP]_Ex_). The levels of PDE3, cGMP concentration and AC activity were varied to see how this affected the steady state concentration of free cAMP in the small compartment, in the presence of diffusion. The AC activity was kept constant (**B**–**E**) in each case and the external cAMP concentration was clamped at 0.4 µM (**B**–**D**), which is similar to the free cAMP concentration obtained in the homogeneous, one-compartment model (Figure 4). The *V*_max_ of PDE3 and the concentration of cGMP (representing NO) were varied and for each pair of values, a steady state cAMP concentration (in compartment) was calculated from the opposing activities of PDE3 and AC (fixed). The *V*_max_ value for PDE3 (1.2 µM^−1^ s^−1^) in the one-compartment model is shown as dotted line (**C**). In (**B**–**D**) the AC activity in the compartment was set to 0.5×, 1.0× and 2× of that used in the one-compartment model (Figure 4, 0.93 µM^−1^ s^−1^), respectively. (**E**) shows the case of elevated external cAMP, e.g. as has been reported by inhibition of PDE2, where the compartment AC activity is set to zero.

The lack of cAMP response to NO in the global cNMP-model and the mimicking of NO effect by PDE3 inhibition could suggest that shape change is governed by events in a rather small compartment enriched in PDE3 and poorer in PDE2. As a first approach, we investigated the cAMP response in a diffusion-coupled compartment containing AC, PDE3 and PKA (Figure 5A). Clamping of the external free cAMP (at the level found in our homogeneous, one-compartment model) and internal free cGMP enabled us to investigate the optimal conditions for PKA activation (Figure 5). It is clearly seen that a cAMP-responsive compartment is feasible at different levels of internal AC activity (Figure 5B–D), as the basal cAMP-level can be kept low at sufficiently high PDE3 levels. However, lower compartment AC activities limit the maximal cAMP level that can be obtained in the presence of diffusion. We also included a situation where a PDE3 compartment is exposed to higher external cAMP levels (e.g. arising from inhibition of PDE2) but at the same diffusion rate constant (Figure 5E). This illustrates several issues; the resistance of the compartment against cAMP overflow at different PDE3 levels, the potential for synergistic communication between two compartments and the possibility of having cAMP-responsive compartments even in absence of AC-activity, given that the basal cAMP-level of the external compartment is relatively high.

### 2.8. A Modest Redistribution of Platelet PDEs Suffice to Generate NO Mediated PKA Activation

**Figure 6:**
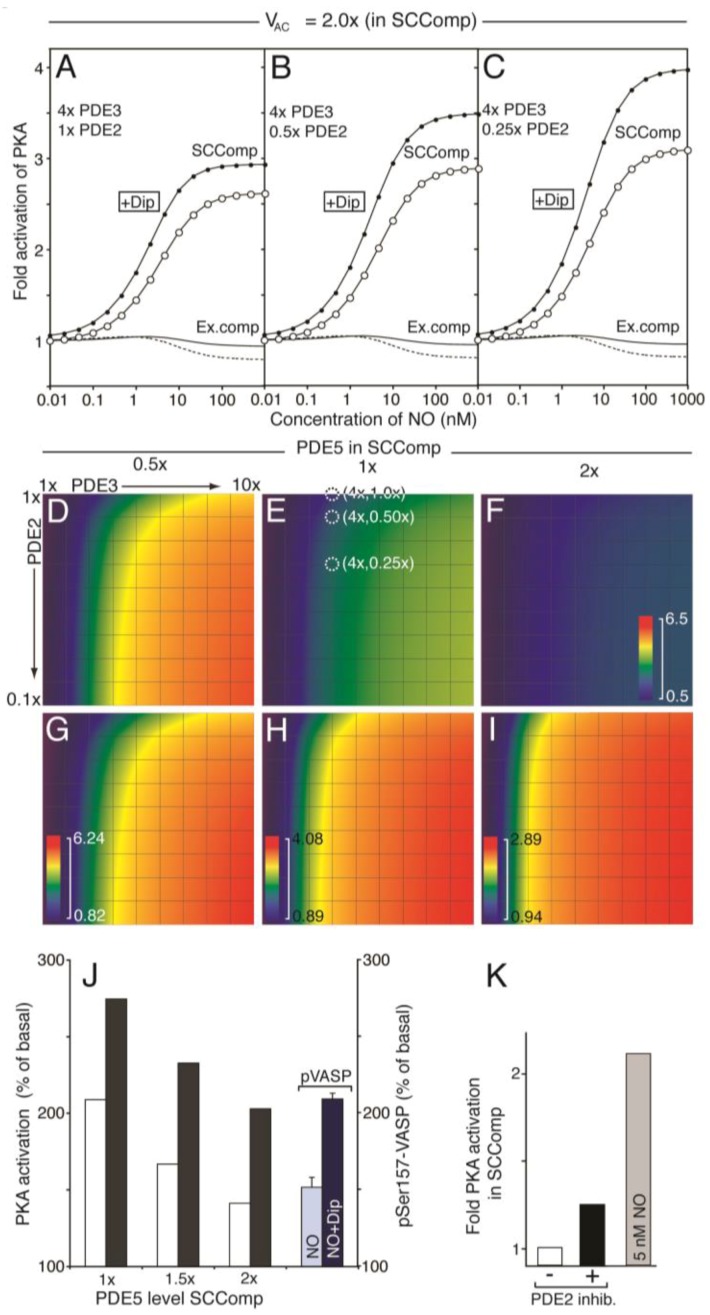
Modelling the effect of PDE redistribution on the PKA response to NO. Starting from the one-compartment model, we investigated how distribution of PDEs influenced cAMP and PKA-activation in a two-compartment model (see supplemental text for details), where a PDE3-rich PKA/VASP compartment (*v_c_*_omp_ = 0.1 × *v*_tot_ = 0.52 fl) was spatially separated from the remaining platelet intracellular volume (*v*_cyt_ 90% of total). The compartment AC concentration was assumed to be two fold that of the one-compartment model (Figure 4, see Results). The level of PDE3 and PDE2 in the compartment was set to n and 1/m fold that of the one-compartment model (Table 1), respectively, keeping the total platelet PDE2, 3 and AC activities as to fulfil experimental cyclic nucleotide measurements. (**A**–**I**) show the PKA response (relative to basal) at different NO concentrations. For (**A**–**C**) the shape change regulating compartment (SCComp) response in absence (○) or presence (●) of dipyridamole (1.0 µM) and the bulk cytosolic (external compartment) response (solid line, dotted line in presence of dipyridamole) was calculated at a steady PDE3 level, varying the PDE2 activity (1×, (**A**); 0.5×, (**B**); 0.25×, (**C**), Table 1). (**D**–**F**) shows the calculated compartment PKA activation in colour code (scale from 0.5 to 6.5 fold activation; (PKA activity at 1 µM NO)/(PKA activity at 0.01 nM NO)), for different distribution of PDE3 and 2. In (**D**) the compartment PDE5 level was set to 0.5 fold the average, whereas in (**E**,**F**) it is set to 1- and 2 fold, respectively. In (**E**), the models that give rise to the compartment PKA activation curve (○) in (**A**) to (**C**) are marked (white dotted circles). (**G**–**I**) shows the same heat diagrams but scaled between their minimal and maximal fold PKA activation. (**J**) shows the compartment PKA response range, at different compartment-levels of PDE5, in the absence (white) or presence (black) of dipyridamole (1.0 μM). The PKA response is shown as the difference between maximal and basal relative to basal (100%). For comparison, we show the steady state levels of Ser157 phosphorylated VASP during NO stimulation in the absence and presence of dipyridamole. (**K**) shows the compartment PKA-activity at basal conditions (white) and during total inhibition of PDE2 (black). For comparison, we show the compartment PKA response to 5 nM NO (grey).

Having established the signalling capacity for a compartment with negligible levels of PDE2, we moved to more realistic models. In particular, since both PDE2 and 3 are considered to be mainly soluble in platelets, even though membrane-bound isoforms and binding partners are known for both PDE isotypes [39,40,41,42,43]. Platelet shape change involves cytoskeletal rearrangements close to the circumferential microtubule band where also the dense tubular system (DTS) of platelets is located. Interestingly, the DTS is rich in platelet AC activity [44]. Based on these experimental findings we constructed a two-compartment model where a postulated small shape change signalling compartment (SCComp, 10% of the platelet volume) had elevated concentration of AC (2 fold, Figure 6). Further, we generated sets of models where the distribution of PDEs between the compartments were different but while keeping other protein species the same and the total PDE activity such as to agree with experimental measurements of total cyclic nucleotide levels.

A modest three-fold increase in PDE3 activity in the SCComp relative to average was enough to facilitate a cAMP-response to NO and this was further increased by a 3-fold decrease in PDE2 level. This was more evident by increasing the compartment PDE3 level to 4-fold (Figure 6A–C), where the effect of reducing PDE2 level on the PKA response is also illustrated. The gain in PKA response was increased most going from 1.0× to 0.5× PDE2 in the SCComp, whereas the dipyridamole response was increased more upon further reduction in PDE2 (Figure 6A–C). The model predicted that while NO/dipyridamole enhanced the PKA activity strongly in the small compartment, the PKA activity decreased in the bulk cytosol. Increased phosphorylation of VASP at the Ser157 PKA site, which blocks nucleation of actin polymerization [45], would therefore be likely to occur in the vicinity of the SCComp.

As PKA can activate PDE3 though phosphorylation, we tested how this could influence the responsiveness of the SCComp. Although we do not know the extent (stoichiometry) of PDE3 phosphorylation upon NO stimulation we estimated an expected extent of PDE3 phosphorylation based on measurements of PDE3 activation and phosphorylation at different cAMP stimuli in platelets. The corresponding expected effect of PDE3 activation based on the PKA activation in the SCComp in response to NO was then included (Figure S3). At the conditions modelled here, it seems that feedback inhibition via PKA phosphorylation of PDE3 was not an important regulatory feature (see supplemental text for more discussion).

Since PDE5 is reported to be partially located in the ER membrane [20], we also investigated the impact of PDE5 concentration on the compartment PKA response (Figure 6 D–I). Here we show the maximal PKA response relative to basal (fold activation; [Csub.] at 1 µM NO/[Csub.] 0.01 nM NO) for a range of models where the PDE distribution between SCComp and the rest of the platelet was varied. Again, for each model the total platelet PDE activities were kept constant. Thus, for three model sets the PDE5-activity of the SCComp was varied to 0.5×, 1× and 2× that of the one compartment model. For each, an array of PKA response values were calculated for different models of PDE3 (1–10×) and PDE2 (1.0–0.1×) SCComp levels compared to the average one-compartment model. Clearly, the compartment activity of PDE5 is an important determinant for the PKA responsiveness at conditions where the PDE3 activity exceeds that of PDE2. Going from 0.5 to 2 fold in compartment PDE5 activity had a large impact on the PKA activation response to NO, which could explain why PDE5 inhibitors have such an effect on NO-inhibited shape change.

We have previously measured the VASP phosphorylation response on the Ser157 PKA-site in the presence and absence of dipyridamole [15]. The fold change observed there could suggest that the PDE5 level in the SCComp was slightly higher than the overall activity (1.5–2×) (Figure 6J). However, this can only be taken as suggestive evidence, as we do not know the abundance of VASP in the predicted SCComp relative to that of entire platelet. We further investigated the SCComp PKA response to total inhibition of PDE2 (Figure 6K), as the latter is known to have little impact on inhibition of platelet shape change experimentally [8]. A modest PKA response was observed to PDE2 inhibition, as compared to a moderate NO stimulation.

## 3. Discussion

Imbalanced cGMP or cAMP signalling causes several diseases of the cardiovascular system. Lowered cAMP signalling in platelets and leukocytes, e.g. due to endothelial dysfunction, is associated with risk of thrombosis and atherosclerosis and in endothelial cells themselves, cAMP is an important regulator of vascular permeability [11]. Cross talk between cGMP and cAMP is functionally implicated in several cell types other than thrombocytes, e.g. beta-adrenergic signalling in cardiomyocytes (for review, see Zaccolo and Movesian [9]) and smooth muscle relaxation [10]. In cardiac myocytes as in platelets, the impact of cGMP on cAMP degradation through PDE2 and 3 can give rise to both inhibitory and cooperative cross-communication of these second messenger systems [46,47]. This makes it particularly interesting to investigate the nature of this signalling communication, also in the perspective of pharmacological targeting and the possibility of off-target effects.

VASP Ser157 phosphorylation is a much used experimental readout for PKA activation. The importance of VASP in platelet NO signalling was clarified in a convincing study by Gawaz and co-authors [48]. Using in vivo microscopy of platelet adhesion to carotid arteries they showed that platelets from VASP knockout mice were unresponsive to NO also in wt recipients. Earlier studies have shown that VASP is important for mediating some of the inhibitory effects of cGMP and cAMP on platelet aggregation, but VASP-deficiency does not affect calcium signalling or secretion [49]. The exact mechanism of VASP action in cells is not fully understood.

Platelet shape change is known to involve reorganization of the circumferential microtubule band, which is important for maintaining the discoid shape of thrombocytes. The dense tubular system (DTS), the residual platelet endoplasmatic reticulum (ER), is associated with the circumferential microtubule band (for review, see White [50]). In addition, the actin cytoskeleton is important for driving extension of pseudopodia and VASP supports the extension of F-actin [51]. It therefore seems reasonable that the relevant signalling compartment is located in the vicinity of the actin and microtubule cortex in association with the DTS. In this region, higher cAMP production can be expected as well as spatial restrictions on the diffusion of the cyclic nucleotides. However, this has to our knowledge not been measured in platelets.

It has remained controversial how moderate changes in cAMP associated with NO stimulation could activate PKA sufficiently. Especially, in light of recent findings on the dissociation of the cAMP-saturated PKA holoenzyme, only moderate activation can be expected and was also found in the modelling (maximal activation of 39% compared to total PKA) even at saturating cAMP [32], due to high levels of PKA in platelets [33]. This has been taken into account in the model presented here. Since it is unlikely that VASP exists at saturating concentrations for both kinase and phosphatase (which could give rise to zero order ultrasensitivity), other mechanisms must come into play [52].

Others and we have pointed to the importance of PDE3A in mediating NO responses in platelets [8,13]. In our model, we make no assumption about the nature of our signalling compartment except for PDE3 activity, which should be present in excess over PDE2 activity, as compared to the remaining platelet volume. In earlier studies of platelet PDE isolation and characterization it was considered that the platelet isotypes were mainly soluble [37]. However, reports that are more recent suggest that this may be an oversimplification, at least for PDE5 [20]. Several isoforms of PDE2 and 3 are known to associate both strongly and dynamically with membranes [42,43]. In addition, protein-protein interaction has been reported for both PDEs [39,41] but is so far not found in association with A-kinase anchoring proteins, which often sequester PDE4 [53]. Thus, a dynamic interaction with membranes for both PDE2 and 3 is possible but is yet to be firmly established in platelets. Interestingly, in cardiac myocytes, membrane associated isoforms of PDE2A and 3A are found in both the plasma membrane and ER, respectively [40,54]. Furthermore, photoaffinity labelling of platelets with [^32^P]-cGMP show evidence for membrane located PDE3A [55]. The low affinity of PDE2 for cGMP and cAMP makes it unsuitable for comparable experiments. In platelets, PDE3A has been found located with the leptin receptor in a signalling complex where AKT activation of PDE3A is postulated to participate in the leptin-induced platelet activation [56].

An interesting finding by the modelling was the influence of PDE5 on PKA activation. Thus, low levels of PDE5 activity enables a larger PKA response to similar NO stimuli. This was intriguing as PDE5 is partially located to the ER membrane, although with a lower specific activity than unbound PDE5 [20]. Studies that quantify PDEs and other cNMP signalling components consistently in platelets would provide highly valuable information for model generation [14,18]. The existing uncertainties make it difficult to conclude on the nature and composition of signalling compartments in platelets as well as in other cells.

The PDE5 inhibitor dipyridamole is favourable in combination with low doses of acetylsalicylic acid (aspirin) as dual antiplatelet therapy for secondary prevention of ischemic stroke [17]. However, dual antiplatelet therapy is not used over extended periods due to high bleeding risk. An alternative strategy may be to potentiate endothelial function. Inhibiting the rate-limiting enzyme in cholesterol biosynthesis (3-hydroxy-3-methylglutaryl-CoA reductase) with statins is found to increase the production of NO in an eNOS-dependent manner [57]. Moreover, additive NO-dependent protective effects of statin and dipyridamole on blood flow and stroke have been reported [58]. Thus, treatment with statins, which increase endothelium-derived NO production and subsequent platelet cGMP production, in combination with PDE5 inhibition by dipyridamole may have a preventative effect on cardiovascular disease without increased risk for bleeding.

A second observation in the models, that does not involve cNMP cross-talk, was the sluggish activation of PKG in platelets, due to sequestration of cGMP at the high affinity site of PKG Iβ and low occupancy of the low affinity site even at saturating NO concentrations. This observation suggests that selective agonists of the low affinity site of PKG Iβ would greatly improve the activation status of PKG in platelets. This could be a targeting option worth testing as an alternative strategy for antiplatelet therapy.

Surprisingly, not much has been done on quantitative modelling of cGMP-cAMP cross talk. To our knowledge this is the first compartment model of cyclic nucleotide signalling in platelets and the first study to investigate the impact of AC and PDE2/PDE3/PDE5 compartmentalization on PKA activation through NO signalling. A similar modelling approach was used to investigate PDE interactions in cardiac β-adrenergic signalling [59]. We believe that this work and similar modelling approaches provide important contributions to the quantitative understanding of cyclic nucleotide signalling and in particular the signalling communication between these second messengers. This is important as the pathways regulate crucial functions in many cell types and tissues. A quantitative understanding of the behaviour of these pathways and their interactions is needed to understand key characteristics that are important for their function. Furthermore, by interrogating such models from different cell types, improved strategies to target cNMP signalling more selectively can be identified, that has minimal off-target effects in other cells or tissues.

## 4. Materials and Methods

### 4.1. Signalling Pathway and Literature Quantitative Information

The active components of the NO-cGMP and cAMP signalling pathways in platelets are well known (Figure 1). In addition, the kinetic characteristics and quantitative information about the network components are reasonably well described (Table 1). All PDE enzymes were modelled with Michaelis Menten kinetics for each of their activity states. Thus, both PDE5 and 2 are activated by binding of cGMP to their GAF domains. PDE2 is also activated by binding of cAMP as well, albeit with a higher dissociation constant. The *K*_D_ values for activation of PDE5 and 2 (Table 1) determine the relative abundance of non-activated and activated enzyme (denoted by * in Table 1) at different concentrations of cGMP and cAMP. We have used different kinetic properties in the activated and non-activated PDEs as for sGC (Table 1). Competitive kinetics was included for all PDEs (Table S1); for PDE2*, cGMP inhibits cAMP degradation (*K_i_* = 22 µM for the activated enzyme); for PDE3, cGMP strongly inhibits cAMP degradation; for PDE5, dipyridamole inhibits cGMP degradation of activated and non-activated enzyme states with the same *K_i_*. The kinetics of PDE2 is described by several Michealis Menten equations to include as much as possible of the kinetic properties reported for this enzyme. Temperature dependency and sensitivity toward type of divalent ion in the assay are important to consider when comparing in vivo and in vitro data. We used the kinetic properties at physiological conditions as reference for our model. The transition between low- and high- activity states, with increased substrate affinities, was used to capture the cooperativity of PDE2. The model showed similar kinetic properties as reported (S_0.5_(cAMP) = 50 µM, Hill 1.3; S_0.5_(cGMP) = 35 µM, Hill 1.1) including a bi-phasic cGMP stimulated cAMP degradation, peaking at about 7 µM cGMP as observed for human platelet PDE2 (Figure S2). For comparison, we included the behaviour of published PDE2 models at similar conditions. Thus, the study of Wangorsch et al. was focused on cAMP signalling and therefore did not include cGMP-modulation of PDE2, as it was not within the scope of the model [14]. For the more recent modelling of heart cAMP and cGMP signalling a PDE2 modelling approach similar to ours was applied [59]. However, for the conditions reported in platelets it did not seem to fit well (Figure S2).

For PKA, the binding affinity of cAMP to the A- and B-site of the holoenzyme is not described. We therefore assumed the affinity for each of the two steps (macroscopic description of binding, i.e., not considering separate binding to the A- or B-site) to be similar, which gives an overall cAMP-binding curve that is in agreement with recently described cAMP-binding to the type-I holoenzyme [31]. The dissociation constant for activation of the cAMP-saturated PKA holoenzyme was set to 1 µM, which is similar to that reported for type II (intermediated between MgATP-and MgADP-bound C-subunit) and for type I at moderate substrate concentrations [32].

### 4.2. Modelling Approach

We have applied a mechanistic modelling approach relying on experimental-based mechanistic and kinetic description of platelet signalling using ordinary differential equations. This is a common approach and has been used in several previous studies on platelets [14,18,19] and assumes well-mixed compartments. Although, platelets have small volumes (5–6 fl), the proteins involved are present at very high levels and it should therefore be valid to not use stochastic differential equations. The proteins involved in the signalling pathways are well described, including the equilibrium binding affinities and the enzyme kinetic constants (Table 1), however, many of the rate constants are not well elucidated. We have therefore mainly focused on the steady state behaviour of the model. The modelling activity was used to quantify contributions to the signalling process coming from different molecular mechanisms at conditions that agree with reported platelet activities and concentrations. Within these frames we further investigated possible mechanisms for compartmentalized signalling, relevant to understand nitric oxide/cGMP mediated cAMP/PKA activation.

The total platelet cyclic nucleotide concentrations were calculated as described (Equations (1)–(6)), taking into account binding of cGMP and cAMP to their respective kinases, as these are present at high concentrations (below). Ordinary differential equations were used to describe changes in cGMP/cAMP (cG/cA) levels (Equations (7)–(10)) as the system evolved to its steady state. Importantly, this does not necessarily mean that the model correctly describes the temporal changes in platelets but it will describe the steady state levels of cNMP and the kinase activities. For the homogeneous one-compartment model (Figure 4), diffusion-mediated transport was ignored. Based on a total platelet volume of 5.2 fl, we assumed an external compartment (4.68 fl) and shape change regulating compartment (SCComp, 0.52 fl) comprising 10% of the total volume [33].

The total level of PKG, 7.30 µM (monomer) and PKA, 3.10 µM (modelled as a dimer of Regulatory and Catalytic subunit, RC), as reported [33]. Binding of cyclic nucleotides to their downstream kinases are denoted by PKG(cG_n_) and R(cA_n_)C for the cGMP- and cAMP-dependent protein kinases, respectively (*n* = 1, 2). Binding of cGMP to PKG-Iβ was modelled as sequential binding of cGMP, first to the high affinity site, second to the low affinity site, due to a 14 fold difference in affinity between the sites. Modelling PKG and PKA as monomers and dimers, respectively, is valid as no interchain interaction is reported for the dimeric PKG and regulatory PKA subunits [60,61].

The kinetics of NO dependent cGMP metabolism in platelets is has been investigated in several studies in rats [4,21,34]. During the first 10 seconds after NO stimulation, a pulsed increase in cGMP is observed before settling at a steady state concentration much lower than the maximal peak concentration (e.g. peak at 300 pmol cGMP/mg protein at 50 nM NO, corresponding to 150 μM cGMP; steady state level <25 pmol/mg) [21]. This pulsed cGMP response is also found in human platelets [4]. The activation of soluble guanylyl cyclase (sGC) was modelled as described, ignoring the time dependent changes, as we were interested in steady state levels [34].

We used the same compartment modelling approach as described previously for cAMP signalling [62,63] and for other signalling pathways [64], where diffusion of free cAMP and cGMP between the compartments is proportional to the concentration difference between them (distribution of proteins and metabolites assumed homogeneous within each compartment). Karpen and co-authors have estimated the exchange flux of cAMP between a membranous compartment and the cytoplasm in HEK 293 cells using a cAMP-responsive ion channel for measuring cAMP concentrations. They reported an exchange rate of 0.8 fl/s, consistent with a diffusion rate of 3 × 10^−6^ cm/s (measured diffusion rate of cAMP in cytoplasm), a barrier length of 1 μm and a cross sectional area of 0.3 μm^2^. Compared to a 40 μm^2^ area expected for their compartment (cubic, 40 fl), we have been much less restrictive in our estimates of barrier length (0.1 μm) and cross sectional area (0.65 μm^2^). However, the diffusion rate of cAMP (3 × 10^−6^ cm^2^/s) can be expected to be reduced in platelets, due to very high levels of cAMP binding sites (at least 6.2 μM [33]). In its bound state, cAMP diffusion would be dramatically decreased or even absent (if PKA is anchored). Similar arguments would hold for cGMP and for simplicity we have set the diffusion flux equal for the two nucleotides.

Assuming a similar apparent diffusion between the compartment and cytoplasm, we calculated a plausible flux (*J*_Diff_) to be 0.44 fl/s (assuming cubic geometry [64,65] 0.52 fl compartment volume, barrier thickness of 0.1 µm), based on 0.8 fl/s for a 40 fl compartment in HEK293 cells (barrier thickness of 1 µm, cAMP diffusion coefficient 3.0 × 10^−6^ cm^2^/s).

(1) $$cG_{tot}^{platelet}=\frac{\upsilon_{cyt}}{\upsilon_{tot}}cG_{tot}^{cyt}+\frac{\upsilon_{comp}}{\upsilon_{tot}}cG_{tot}^{comp}$$

(2) $$cG_{tot}^{cyt}=cG_{bound}^{cyt}+cG_{free}^{cyt};cG_{tot}^{comp}=cG_{free}^{comp}+cG_{free}^{comp}$$

(3) $$cA_{tot}^{platelet}=\frac{\upsilon_{cyt}}{\upsilon_{tot}}cA_{tot}^{cyt}+\frac{\upsilon_{comp}}{\upsilon_{tot}}cA_{tot}^{comp}$$

(4) $$cA_{tot}^{cyt}=cA_{bound}^{cyt}+cA_{free}^{cyt};cA_{tot}^{comp}=cA_{bound}^{comp}+cA_{free}^{comp}$$

(5) $$cG_{bound}=PKG(cG)+2\cdot PKG(cG_{2})$$

(6) $$cA_{bound}=R(cA)C+2\cdot R(cA_{2})C+2\cdot R(cA_{2})$$

(7) $$\frac{d}{dt}cG_{free}^{cyt}=V_{GC}^{cyt}-V_{PDE5}^{cyt}-V_{PDE2,cG}^{cyt}-\frac{J_{Diff}}{\upsilon_{cyt}}(cG_{free}^{cyt}-cG_{free}^{comp})$$

(8) $$\frac{d}{dt}cG_{free}^{comp}=V_{GC}^{comp}-V_{PDE5}^{comp}-V_{PDE2,cG}^{comp}+\frac{J_{Diff}}{\upsilon_{cyt}}(cG_{free}^{cyt}-cG_{free}^{comp})$$

(9) $$\frac{d}{dt}cA_{free}^{cyt}=V_{AC}^{cyt}-V_{PDE3}^{cyt}-V_{PDE2}^{cyt}-\frac{J_{Diff}}{\upsilon_{cyt}}(cA_{free}^{cyt}-cA_{free}^{comp})$$

(10) $$\frac{d}{dt}cA_{free}^{comp}=V_{AC}^{comp}-V_{PDE3}^{comp}-V_{PDE2}^{comp}+\frac{J_{Diff}}{\upsilon_{comp}}(cA_{free}^{cyt}-cA_{free}^{comp})$$

(11) $$\frac{k_{dephos}}{k_{phos}}=[Kinase*]\frac{1-S_{pPDE}}{S_{pPDE}}$$

where the superscript refers to the compartment (comp—shape change regulated compartment (SCComp), cyt—external compartment) and subscript to the specific enzyme for rates and state (bound or unbound/free) for metabolites. For PDE2, which has two substrates, this is also specified in the subscript. Reaction rates are specified with *V*, volumes with υ and the diffusion flux with *J*_Diff_. Equation (11) describes the steady state relationship between the ratio of dephosphorylation and phosphorylation rate constants as a function of the concentration of active kinase ([Kinase*]) and the observed phosphorylation stoichiometry of PDE (*S*_pPDE_). Thus, rates of phosphorylation (*V*_phos_ = *k*_phos_[PDE][Kinase*]) and dephosphorylation (*V*_dephos_ = *k*_dephos_[pPDE]) are represented by linear kinetics (assuming high *K*_m_ values).

### 4.3. Parameter Estimation

In this study, we have mainly relied on the quantitative measurements of sGC and cGMP-PDE activities performed on human platelets [4]. Here maximal peak cGMP concentrations of about 60 µM and maximal steady state values below 10 µM were observed (in absence of PDE inhibitors). Also here, steady state was established well within one minute, which is typically used here. The *V*_max_ for sGC was set to 20 µM/s as measured in human platelets [4], whereas the basal sGC activity (*k*_cat_) was fitted to about 1/400 of the value for the activated enzyme, which is similar to findings on the recombinant enzyme [24]. Adjustments of the PDE V_max_ activities were performed to fit measured cAMP and cGMP levels [16], keeping total cAMP PDE activity close to 2 µM s^−1^ as reported at 1 µM substrate [66] and AC activity < 1 µM s^−1^. A constant adenylate cyclase (AC) activity was assumed. The fitting was also restricted by PDE2 activity being less than 10% of the PDE5 activity at 1 µM cGMP [66].

### 4.4. Simulations

The model was implemented in COPASI 4.11 (build 65, www.copasi.org), which uses an LSODA ODE-solver for stiff and non-stiff systems [67,68]. The absolute tolerance was set to 1.0 × 10^−12^. The models will be deposited in the biomodels.org repository.

## 5. Conclusions

We have performed an analysis of cGMP to cAMP cross-talk in platelets using a mechanistic modelling approach. The modelling showed the importance of including cNMP binding to intracellular receptors to correctly balance the activities of cyclases and phosphodiesterases and to assess the potency of competitive pharmacological inhibitors. This is particularly evident in platelets, which contain extreme levels of such sequestering receptors. Compartment models with heterogeneous distribution of phosphodiesterases could account for experimental observations on NO mediated PKA activation. The compartment models revealed an interesting relationship between the different phosphodiesterases in platelets, which may be linked to the observed clinical efficiency of PDE inhibitors. Further experimental evidence is required to identify the nature and composition of signalling compartments in platelets. This is likely to provide valuable knowledge for the development of novel antiplatelet therapies.

## Figures and Tables

**Table 1:** Description of model parameters. The kinetic parameters used in the different models are shown below. For the two compartment models, the *V*_max_ values were varied as described in the figures. For models where PDE phosphorylation was incorporated ([PDE5]_tot_ 1.0 µM, [PDE3]_tot_ 1.2 µM), the *V*_max_ values were refitted to experimental cNMP values. [GTP] fixed at 600 µM.

Enzymes	Reactions	Parameters	Ref.
sGC	sGC + NO ⟺ sGC:NO	*k*_f_ = 300 µM^−1^ s^−1^, *k*_b_ = 14 s^−1^ *K*_1_ = *k*_b_/*k*_f_ = 46.7 nM	[23]
	sGC:NO ⟺ sGC* ^1^	*k*_f_ = 1000 s^−1^, *k*_b_ = 280 s^−1^ *K*_2_ = *k*_b_/*k*_f_ = 0.28	[23]
	GTP ⟶ cG ^3^	$V_{\max}^{sGC}$^2^ = 0.05, $K_{m}^{sGC}$= 100 µM	[4,24]
sGC*	GTP ⟶ cG	$V_{\max}^{sGC*}$= 20, $K_{m}^{sGC*}$= 50 µM	[4]
PDE5/pPDE5 ^4^	PDE5* ⟺ PDE5 + cG	$K_{D}^{PDE5}$= 130 nM	[7]
	pPDE5* ⟺ pPDE5 + cG	$K_{D}^{pPDE5}$= 30 nM	[7]
	cG ⟶ GMP	$V_{\max}^{PDE5}$= 39.0, $K_{m}^{PDE5}$= 4.60 µM, $K_{i}^{PDE5}$= 0.70 µM	[5,6,25]
PDE5*/pPDE5*	cG ⟶ GMP	$V_{\max}^{PDE5*}$= 117, $K_{m}^{PDE5*}$= 1.00 µM, $K_{i}^{PDE5*}$= 0.70 µM	
PDE2	PDE2* ⟺ PDE2 + cG	$K_{D}^{PDE2}$= 2.00 µM	[26]
	PDE2* ⟺ PDE2 + cA	$K_{D}^{PDE2}$= 25.0 µM	[26]
	cG ⟶ GMP	$V_{\max,cG}^{PDE2}$= 24.0, $K_{m,cG}^{PDE2}$= 120 µM	[27,28]
PDE2*	cG ⟶ GMP	$V_{\max,cG}^{PDE2*}$= 240, $K_{m,cG}^{PDE2*}$= 15.0 µM	[27,28]
PDE2	cA ^5^ ⟶ AMP	$V_{\max,cA}^{PDE2}$= 24.0, $K_{m,cA}^{PDE2}$= 120 µM, $K_{i}^{PDE2}$= 120 µM	[27,28]
PDE2*	cA ⟶ AMP	$V_{\max,cA}^{PDE2*}$= 240, $K_{m,cA}^{PDE2*}$= 20.0 µM, $K_{i}^{PDE2*}$= 22.0 µM	[27,28]
PDE3/pPDE3 ^6^	cA ⟶ AMP	$V_{\max}^{PDE3}$= 1.2, $K_{m}^{PDE3}$= 150 nM, $K_{i}^{PDE3}$= 60.0 nM	[29]
AC	⟶ cA	0.93 µM^−1^s^−1^	
PKG	PKG(cG) ⟺ PKG + cG	*K*_D_ = 55.0 nM	[30]
	PKG(cG_2_) ⟺ PKG(cG) + cG	*K*_D_ = 750 nM	[30]
PKA	R(cA)C ⟺ RC + cA	*K*_D_ = 2.90 µM	[31]
	R(cA_2_)C ⟺ R(cA)C + cA	*K*_D_ = 2.90 µM	[31]
	R(cA_2_)C ⟺ R(cA_2_) + C	*K*_D_ = 1.00 µM	[32]

^1^ Activated enzyme forms are indicated (*), ^2^ *V*_max_ values are given as µMs^−1^, ^3^ cGMP abbreviated cG, ^4^ phosphorylated PDE5 has higher affinity for cGMP binding to the GAF A domain (*K*_D_ 30 nM) and slightly higher *V*_max_ (1.2 fold), ^5^ cAMP abbreviated cA, ^6^ phosphorylation of PDE3 increases its maximal activity 3 fold.

## Supplementary Materials

Supplementary materials can be found at http://www.mdpi.com/1422-0067/19/2/612/s1.
