# Supplementary material for: Mathematical Modelling of Nitric Oxide/Cyclic GMP/Cyclic AMP Signalling in Platelets
Source: Int J Mol Sci. 2018 Feb 19;19(2):612. doi: 10.3390/ijms19020612 (PMC5855834; doi:10.3390/ijms19020612)
Supplement: Supplementary file 1 [file ijms-19-00612-s001.pdf]

Supplemental Discussion

PKA-mediated phosphorylation of PDE3 is reported to increase its activity in platelets and other cells. Since the PKA-mediated phosphorylation and activation of PDE3A during NO stimulation is not described qualitatively or quantitatively it was not included in our model. The impact of this feedback inhibition will be to lower PKA activation, hence be counterproductive for the compartment PKA response to NO. However, at conditions of strong PKA activation, it provides a mechanism for restraining platelet antagonistic responses. Phosphorylation of PDE3A is reported by several kinases in platelets. Thus, PKA, PKC and PKB all phosphorylate and activate PDE3A, albeit at different residues. Unlike PKC phosphorylation, the PKA phosphorylation site do not induce binding of the adaptor proteins 14-3-3, which have been shown to prolong protein phosphorylation through competitive inhibition of phosphatases [1,2]. In a recent study, PDE3A phosphorylation by different kinases was studied in platelets, showing that cAMP stimulation by forskolin, 8-CPT-cAMP or PGE1 resulted in a 40 % increase in PDE3A activity [3]. At such stimuli, a cAMP level of >20 M has been found, which enables an estimation of PKA activation levels [4,5]. We have used this information to correct for a realistic level of PDE3 phosphorylation in our two-compartment NO signaling model (Fig. S2). At the PKA activation levels we find in our model, the impact of this phosphorylation seems to be modest. However, as little is known about the distribution of PDE3 in platelets, less is known about possible spatial differences in PDE3 phosphorylation in platelets. In conclusion, we expect PDE phosphorylation to have larger impact at conditions where cAMP-levels increase more dramatically and feedback inhibition serve as an efficient way of lowering platelet inhibition during strong agonistic stimulation.

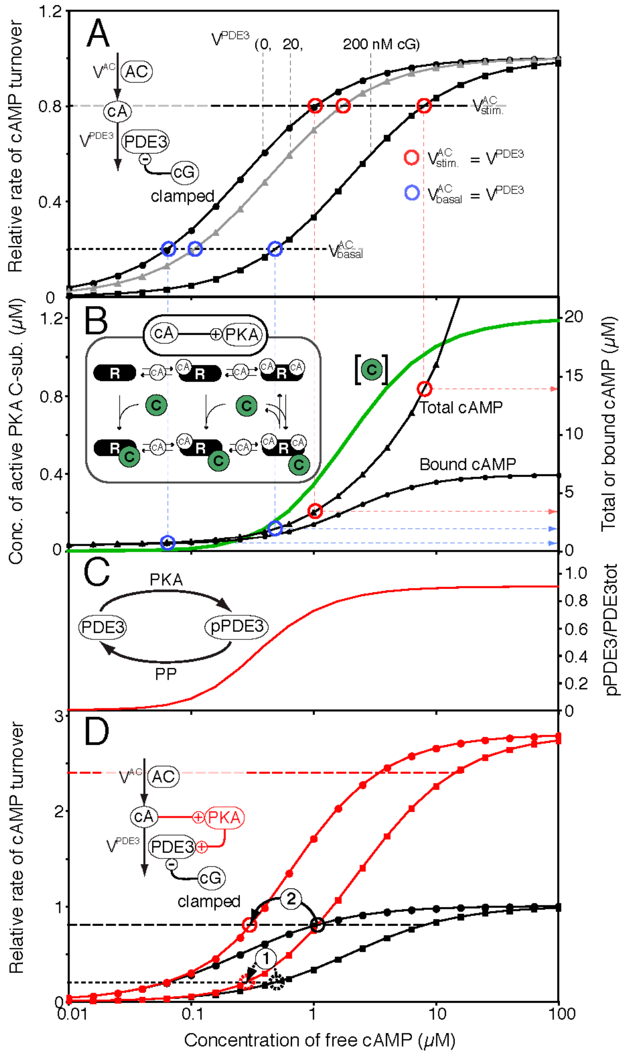

Figure S1. PDE3 kinetics and its modulation by cGMP and PKA phosphorylation. (**A**) illustrates a cell where the concentration of free cAMP is governed by the activity of adenylate cyclase (AC, V^{AC}) shown as horizontal dotted lines for basal AC activity ($V^{AC}{}_{basal}$) and stimulated AC activity ($V^{AC}{}_{stim}$). The activity of PDE3 (V_{max}, normalized to 1) is shown as a function of the concentration of free cAMP and in the presence of 0 (●), 20 (▲) or 200 (■) nM cGMP. The steady state cAMP level of such a cell at basal or stimulated AC levels and for different cGMP levels is shown as blue or red circles, respectively. (**B**) shows the activation of PKA in response to cAMP, using the same activation kinetics as for the rest of the study (Table 1). The concentration of free catalytic subunit ([C], green line) as well as the total cellular cAMP levels (▲) and the PKA-bound cAMP levels (●), shown on the right axes. (**C**) using linear phosphorylation/dephosphorylation kinetics ($v_{phos} = k_{phos}$*[PDE3]*[C], $v_{dephos} = k_{dephos}$*[pPDE3], $k_{phos} = 0.2811\ \mu M^{-1}s^{-1}$, $k_{dephos} = 1\ s^{-1}$) we modeled the phosphorylation of PDE3 in response to PKA activation at conditions giving 90 % phosphorylation stoichiometry of PDE3 at maximal PKA activation. (**D**) shows the effect of PKA mediated PDE3 phosphorylation at conditions where up to 90 % phospho-PDE3 can be generated (very strong feedback inhibition) (red curves) compared to the PDE3 activity without PKA mediated phosphorylation (black lines). The PDE3 activities at 0 (●) or 200 nM (■) cGMP is shown and the conditions of two AC activity levels are illustrated where for the low (1) and medium (2) AC activity the left-shift in steady state cAMP concentration upon introduction of feedback inhibition is illustrated by arrows.

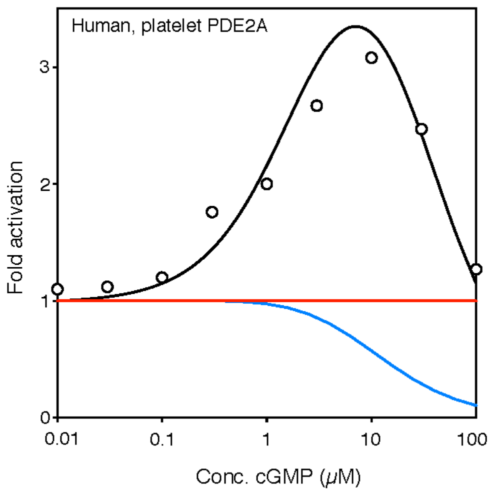

Figure S2. PDE2 models and fit to data from human platelet PDE2. Based on data on human platelet PDE2 from Grant et al. [6] (O) we tested the performance of two published PDE2 models; Wangorsch et al. [7] (red line) and Zhao et al. [8] (blue line) with the model used in our study (black line). The model by Wangorsch et al. was as we understand it not constructed to account for PDE2 response to cGMP, as cAMP was the focus of the study.

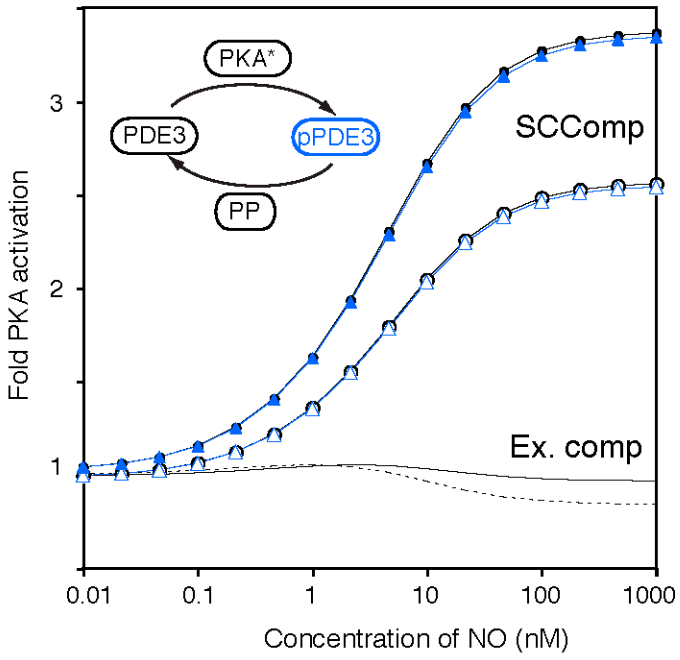

Figure S3. Effect of PKA mediated PDE3 phosphorylation on compartment response to NO. According to recent observation on PDE3 activation in platelets, stimulation of platelets with strongly PKA activating agents (forskolin, 8-CPT-cAMP, or PGE1) resulted in 40% activation of PDE3 [3]. This corresponds to a maximal 20% phosphorylation of PDE3 (3 fold increase in V_{max} for PDE3 upon phosphorylation), at these conditions where platelet cAMP levels > 20 M can be expected [5]. From this information we calculated the impact of PKA mediated PDE3 phosphorylation and activation on the PKA response of the two-compartment model (4xPDE3, 0.25xPDE2). The original model is shown in black and the model with PDE3 phosphorylation in blue. Open/closed symbols and solid/dotted line denote absence/presence of dipyridamole, respectively.

References

1. Kleppe, R.; Toska, K.; Haavik, J. Interaction of phosphorylated tyrosine hydroxylase with 14-3-3 proteins: evidence for a phosphoserine 40-dependent association. *J Neurochem* **2001**, *77*, 1097-1107.
2. Palmer, D.; Jimmo, S.L.; Raymond, D.R.; Wilson, L.S.; Carter, R.L.; Maurice, D.H. Protein kinase A phosphorylation of human phosphodiesterase 3B promotes 14-3-3 protein binding and inhibits phosphatase-catalyzed inactivation. *J Biol Chem* **2007**, *282*, 9411-9419.
3. Hunter, R.W.; Mackintosh, C.; Hers, I. Protein kinase C-mediated phosphorylation and activation of PDE3A regulate cAMP levels in human platelets. *J Biol Chem* **2009**, *284*, 12339-12348.
4. Eigenthaler, M.; Nolte, C.; Halbrugge, M.; Walter, U. Concentration and regulation of cyclic nucleotides, cyclic-nucleotide-dependent protein kinases and one of their major substrates in human platelets. Estimating the rate of cAMP-regulated and cGMP-regulated protein phosphorylation in intact cells. *Eur J Biochem* **1992**, *205*, 471-481.
5. Halbrugge, M.; Friedrich, C.; Eigenthaler, M.; Schanzenbacher, P.; Walter, U. Stoichiometric and reversible phosphorylation of a 46-kDa protein in human platelets in response to cGMP- and cAMP-elevating vasodilators. *J Biol Chem* **1990**, *265*, 3088-3093.
6. Grant, P.G.; Mannarino, A.F.; Colman, R.W. Purification and characterization of a cyclic GMP-stimulated cyclic nucleotide phosphodiesterase from the cytosol of human platelets. *Thromb Res* **1990**, *59*, 105-119.
7. Wangorsch, G.; Butt, E.; Mark, R.; Hubertus, K.; Geiger, J.; Dandekar, T.; Dittrich, M. Time-resolved in silico modeling of fine-tuned cAMP signaling in platelets: feedback loops, titrated phosphorylations and pharmacological modulation. *BMC Syst Biol* **2011**, *5*, 178.
8. Zhao, C.Y.; Greenstein, J.L.; Winslow, R.L. Interaction between phosphodiesterases in the regulation of the cardiac beta-adrenergic pathway. *J Mol Cell Cardiol* **2015**, *88*, 29-38.
